# Apologetic

**Published:** 1897-08

**Authors:** 


APOLOGETIC: When a "medical editor," who uses such expressions in his editorials as "we expected to make some of *these* kind of folks mad," and "we have not and we will not do any such thing,"* [we have not *do*?] applies to representative members of the medical profession, who have been honored by the State Medical Association, such epithets as "mountebanks and adventurers," † because they want to establish a medical college in opposition to said editor's views, his utterances must be taken with a large grain of allowance.

*Texas Medical Practitioner for July, Page 10.
†Texas Medical Practitioner for July, Page 23.

